# Exploring psychedelic-assisted therapy in the treatment of functional seizures: A review of underlying mechanisms and associated brain networks

**DOI:** 10.1177/02698811241248395

**Published:** 2024-04-23

**Authors:** Evan Cole Lewis, Alexandria Jaeger, Manesh Girn, Egiroh Omene, Madeline Brendle, Elena Argento

**Affiliations:** 1 Hospital for Sick Children, Department of Pediatrics, University of Toronto, Toronto, ON, Canada; 2 Numinus Wellness Inc., Vancouver, BC, Canada; 3 Neuroscape, Department of Neurology, University of California San Francisco, San Francisco, CA, USA; 4 Health Outcomes Division, College of Pharmacy, University of Texas at Austin, Austin, TX, USA; 5 Department of Psychology, University of British Columbia, Kelowna, BC, Canada

**Keywords:** Functional seizures, psychedelic-assisted therapy, functional neurological disorder, psychedelics, psychogenic non-epileptic seizures

## Abstract

Functional seizures (FS), the most common subtype of functional neurological disorder (FND), cause serious neurological disability and significantly impact quality of life. Characterized by episodic disturbances of functioning that resemble epileptic seizures, FS coincide with multiple comorbidities and are treated poorly by existing approaches. Novel treatment approaches are sorely needed. Notably, mounting evidence supports the safety and efficacy of psychedelic-assisted therapy (PAT) for several psychiatric conditions, motivating investigations into whether this efficacy also extends to neurological disorders. Here, we synthesize past empirical findings and frameworks to construct a biopsychosocial mechanistic argument for the potential of PAT as a treatment for FS. In doing so, we highlight FS as a well-defined cohort to further understand the large-scale neural mechanisms underpinning PAT. Our synthesis is guided by a complexity science perspective which we contend can afford unique mechanistic insight into both FS and PAT, as well as help bridge these two domains. We also leverage this perspective to propose a novel analytic roadmap to identify markers of FS diagnostic specificity and treatment success. This endeavor continues the effort to bridge clinical neurology with psychedelic medicine and helps pave the way for a new field of psychedelic neurology.

## Introduction

Functional neurological disorder (FND) is one of the most common conditions presenting to neurology clinics, posing a significant burden of disability and distress (Espay et al., 2018). FND can take several forms, including functional movement disorders, sensory disorders, and functional seizures (FS) (Drane et al., 2020; Lidstone et al., 2020). FS are one of the most common presentations of FND (Aybek and Perez, 2022) and impose a substantial economic burden on healthcare systems, contributing to the overall costs of FND estimated at $1.2 billion per year in the USA (Seneviratne et al., 2019; Stephen et al., 2021). Sometimes referred to as dissociative seizures, non-epileptic attack disorder, or psychogenic nonepileptic seizures, FS are characterized by episodic disturbances of normal functioning and reduced self-control that manifest in events with similarities to epileptic seizures (Brown and Reuber, 2016b). Rates of post-traumatic stress disorder (PTSD), anxiety, and depression are higher in those with FS than the general population and those with epilepsy, and 75% of diagnosed individuals are women (Goldstein et al., 2020; Hallett et al., 2022). Risk factors for FS are multifactorial and include psychosocial comorbidities such as emotional stressors and a history of childhood adversity, neglect, or trauma (Baslet, 2011; Brown and Reuber, 2016b; Goleva et al., 2020; Hallett et al., 2022). Although epidemiological data on FS are limited, incidence and prevalence rates have been recently estimated at 3.1/100,000 and 23.8/100,000 per year, respectively, approaching the prevalence of multiple sclerosis (Villagrán et al., 2021).

FS presents a challenge due to the absence of consistent diagnostic and treatment approaches, which, in turn, result in the absence of a standardized care pathway. Misdiagnosis, delayed diagnosis, and prescriptions for improper medications, such as antiseizure medication, tend to worsen symptoms (Bennett et al., 2021; LaFrance et al., 2014). Psychological interventions, such as cognitive behavioral therapy (CBT)-informed psychotherapy, are considered first-line treatment; however, supportive evidence remains limited, as does their availability (Carlson and Nicholson Perry, 2017; Goldstein et al., 2020). In addition, although several models have been proposed to explain the mechanisms of FS, none provide a complete account of the condition or its underlying cause (Brown and Reuber, 2016b; Hallett et al., 2022). Neuroimaging data have revealed a heterogeneous set of findings that implicate dysfunction across multiple large-scale networks, with connectivity abnormalities involving networks associated with emotional processing, attention, interoception, self-agency, and motor control (Aybek and Perez, 2022; Balachandran et al., 2021; Ertan et al., 2022; Sojka et al., 2022; Szaflarski and LaFrance, 2018). The limitations of current diagnostic and treatment approaches, compounded by the lack of a unified mechanistic model, highlight the need for further research and exploration of novel interventions.

A novel intervention with potential relevance for treating FS is psychedelic-assisted therapy (PAT). The term “psychedelic” typically refers to the so-called “classic” or serotonergic psychedelic drugs, which include lysergic acid diethylamide (LSD), psilocybin, N,N-dimethyltryptamine/ayahuasca, and mescaline. These drugs all elicit potent and wide-ranging subjective effects via agonism and partial agonism at the 5-HT2A receptor (Kwan et al., 2022; Nichols, 2016; Vollenweider and Preller, 2020). A broader definition—which we adopt here—also includes ketamine, a “dissociative anesthetic” with dose-dependent psychedelic-like qualities, and the “entactogen” or “empathogen,” 3,4-methylenedioxymethamphetamine (MDMA). A large and growing body of evidence supports the safety and efficacy of PAT for treating a wide range of mental health conditions, including mood disorders, substance use disorders, and PTSD (Dunlap et al., 2018; Mitchell et al., 2021). A variety of clinical trials highlighting PAT efficacy for various indications including PTSD, OCD, anxiety, and depression are summarized in Supplemental Table S1. This apparent transdiagnostic efficacy has motivated investigations of PAT in treating additional psychiatric and neurological disorders, including FND (Stewart et al., 2020). A small but growing number of studies, patient surveys, and reviews have been published exploring the role of psychedelics as a treatment for FND. These have been motivated by (i) FND’s close relationship to mental health conditions such as depression, anxiety, and PTSD, (ii) the high incidence of patient-reported psychedelic self-treatment for this class of disorders, and (iii) mechanistically based arguments suggesting a match between psychedelic therapeutic effects and FND pathophysiology (Butler et al., 2020, 2023; Stewart et al., 2020; Vendrell-Serres et al., 2021). A systematic review of earlier research conducted in the 1950s and 1960s investigating psychedelics for treating FND (primarily with LSD) found that of the 26 patients described in the case series and reports, 69% (*n* = 18) experienced some improvements, with 23% (*n* = 6) having made complete recoveries (Butler et al., 2020). Two recent case reports signal promise. One describes the successful treatment of a patient with FNDs, characterized by sensory and motor paralysis of the left arm, using intranasal esketamine, the S-enantiomer of ketamine (Vendrell-Serres et al., 2021). The other, conducted by members of our team, describes the first case of a patient who experienced significant reductions in the severity and frequency of FS, as well as marked improvements in depressive symptoms and well-being, following sublingual and intranasal ketamine-assisted therapy (KAT) (Argento et al., 2023). Finally, a recent study assessed self-management strategies and views on novel treatments for FND, including psychedelics, with an online, cross-sectional survey of patients with FND recruited through social media and patient groups internationally (Butler et al., 2023). The authors reported that 15% of the 980 respondents had used illicit substances (i.e., cannabis, cocaine, psychedelics) that were moderately effective. These preliminary findings encourage further investigation into the therapeutic potential of psychedelics in the context of FND and subtypes such as FS.

Primary molecular targets and consequent intracellular signaling cascades vary across psychedelic substances, with potential convergence on plasticity-based pathways which are an active area of current research (Carhart-Harris, 2019; Nardou et al., 2023). Emerging perspectives suggest that psychedelics may induce acute and post-acute pro-plasticity effects that, when combined with psychological support and positive contextual factors, facilitate enhanced learning and a revision of internal models improving mental health and well-being (Brouwer and Carhart-Harris, 2021; Carhart-Harris and Friston, 2019; Carhart-Harris et al., 2023; Nardou et al., 2019, 2023). Human neuroimaging research with psychedelics has demonstrated significant alterations to functional connectivity (FC) within and between large-scale brain networks during the acute psychedelic experience (Girn et al., 2023; Ionescu et al., 2018; McCulloch et al., 2022; Müller et al., 2018, 2021), which may partially persist post-acutely with relevance to therapeutic outcomes (Daws et al., 2022). Emerging work on serotonergic psychedelics and ketamine has unveiled a complex mosaic of FC changes indicative of increased whole-brain integration, the precise details of which are relatively inconsistent across studies (Girn et al., 2023; Ionescu et al., 2018; McCulloch et al., 2022; Müller et al., 2018). With respect to MDMA, only two human neuroimaging studies have been conducted, but findings also suggest an intricate and variable set of large-scale network changes (Carhart-Harris et al., 2015; Müller et al., 2021).

A recently proposed framework sought to reconcile the apparent inconsistencies in psychedelic neuroimaging by providing a perspective informed by complexity science (Girn et al., 2023). According to this perspective, rather than seeking to identify a representative pattern of large-scale network changes related to a particular pathology, drug, or intervention, the authors suggest examining properties pertaining to how the brain dynamically traverses different patterns or states over time as an integrated system of interacting parts. This perspective intuitively explains several aspects of psychedelic effects, including their inter-individual variability and sensitivity to contextual factors, and renders previous apparently inconsistent findings more intelligible under a unified framework. Given similar inter-study and inter-subject variability in large-scale network abnormalities observed in the FS functional neuroimaging literature, it is possible that applying a complex system perspective may help reconcile differences there as well. This may, in turn, provide observations that are clearer markers of FS diagnosis and treatment success, which could be utilized as biomarkers in future studies.

This paper proposes that FS represents a well-defined cohort to study the large-scale neural mechanisms underpinning the therapeutic efficacy of PAT. In this article, we explore the theoretical and mechanistic framework of PAT as a potential treatment for FS and propose a roadmap by which approaches informed by complexity science could be applied to treatment responders in a future clinical trial before and after treatment, such that markers of diagnostic specificity and treatment success can be identified. This would provide an opportunity for an alternative treatment in a population of individuals who historically carry a poor prognosis, in addition to informing our understanding of the mechanisms that underlie psychedelic drug action. Furthermore, this expanded breadth of psychedelic applications paves the way for greater synthesis between the disciplines of neurology and psychiatry, transcending diagnostic and disciplinary boundaries and potentially offering greater hope for patients with complex conditions and medically unexplained symptoms.

## FS and brain networks

### Mechanistic models of FS

FS are complex and not fully understood, but various theoretical models have been proposed to explain them (Brown and Reuber, 2016a). These models hinge on a conceptualization that FS serve as a release mechanism for trauma or psychological distress (Harden, 1997). Historically, psychological concepts like hysteria and conversion were used to address medically unexplained symptoms, including FS. Recent models propose hard-wired responses, dysregulated affective processing, or learned behaviors as contributing factors (Brown and Reuber, 2016a; Roberts and Reuber, 2014). The hard-wired response model suggests that FS are reflex-like, involuntary responses to perceived threats that are akin to defensive reflexes like freezing (Brown and Reuber, 2016a). By contrast, the dissociative model posits that FS occur because of a dissociative disintegration of consciousness, memory, and perception in response to experiences of overwhelming stress or trauma (Roberts and Reuber, 2014). Lastly, the learned behavior model is based on the finding that FS are more likely to occur after an individual has witnessed or learned about seizures (Reuber and Brown, 2017). According to this model, FS first develop as a result of classical conditioning and are then reinforced and perpetuated by operant conditioning (i.e., due to their ability to temporarily relieve symptoms of distress) (Hallett et al., 2022; van der Kruijs et al., 2012, 2014).

The so-called “integrative cognitive model” (ICM) has been proposed to unify these models and provide a more coherent framework for understanding the mechanisms underlying FS (Reuber and Brown, 2017). This model, introduced by Reuber and Brown in 2017, posits that FS result from the interaction of biological, psychological, and social factors that predispose, precipitate, and perpetuate the disorder (Reuber and Brown, 2017; Sojka et al., 2022). According to the ICM, the observable and subjective aspects of FS arise from the automatic enactment of a learned representation called the “seizure scaffold.” This seizure scaffold is shaped by intrinsic reflexes, physical symptoms (such as syncope, dissociation, and head injury), and knowledge or imitation of seizure-like behavior—together synthesizing the major factors described in previous models. Triggering of the scaffold can be brought about by various stimuli, including autonomic arousal and stress, as well as internal and external cues such as conditioned negative emotions or thoughts and trauma reminders. These triggers typically occur in the context of compromised inhibition of emotional processing/interoceptive awareness by higher-level cortical areas. Ultimately, the ICM proposes that FS serve as an adaptive regulatory mechanism to attenuate states of hyperarousal within the individual. This is consistent with findings indicating states of chronically elevated arousal in patients with FS despite a lack of conscious awareness (i.e., “panic without panic”), as evidenced by objective markers of increased sympathetic and decreased parasympathetic tone (van der Kruijs et al., 2016).

Symptoms of FS relate to neuropsychological constructs that map onto morphological and functional abnormalities within large-scale networks underpinning cognitive, emotional, attentional, and sensorimotor processes (Drane et al., 2020). As shown in Table 1, functional neuroimaging studies of patients with FS demonstrate multi-focal aberrations spanning several large-scale brain regions and networks. These observed network dysfunctions have the potential to provide a biological framework that accommodates the major factors described by the ICM and is supported elsewhere (Sojka et al., 2022). However, studies to date have yielded heterogeneous findings that appear, at first glance, to be somewhat inconsistent, increases and decreases in activation of the rTPJ, cortical thickness, reactivity in the hippocampus, reactivity, and connectivity in the amygdala—likely reflecting a combination of the nonspecific limitations of functional neuroimaging studies (Girn et al., 2023) and the clinical diversity of FS patients (Table 1).

**Table 1.** Brain region and network abnormalities associated with FS.

Brain region	Key findings	Methods	Cohort and confounding trauma	Studies
DMN	↓ In cortical thickness ↓ White matter connectivity ↑ Coactivation of the precuneus and cingulate gyri ↓ Coactivation of the precuneus and sensorimotor cortex	DTI, MRI, PET, SPECT, fMRI	FS and no co-existing psychiatric conditions; FS with mixed psychiatric conditions	• McSweeney et al. (2017) • Sone et al. (2019) • van der Kruijs et al. (2014) • Sone (2023)
PFC (executive control)	↑ Reactivity in dmPFC ↓ Reactivity in dlPFC	fMRI	TBI compared with TBI + FS	• Balachandran et al. (2021)
Medial temporal (emotional processing)	↑ Reactivity in the right hippocampus ↓ Left hippocampus, cingulum, and amygdala ↓ Reactivity in the amygdala and left hippocampus ↑ Resting-state functional connectivity in the right amygdala, motor cortex, and middle frontal gyrus ↑ Propagation between insula, parahippocampus, hippocampus, putamen ↑ Coactivation of the cingulate, insula, and motor cortex	fMRI	TBI compared with TBI + FS; FS with mixed psychiatric conditions; FS and no co-existing psychiatric conditions	• Balachandran et al. (2021) • Allendorfer et al. (2019) • van der Kruijs et al. (2014)
Sensorimotor cortex	↑ Cortical thickness in motor and sensory areas	MRI	FS and no co-existing psychiatric conditions *controls not excluded for psychiatric conditions	• McSweeney et al. (2018)
Motor Cortex	↑ Connectivity from M1 to SMA, MCC, TPJ, dmPFC, and putamen ↑ Connectivity from motor cortex to amygdala and insula, positively correlated with physical abuse severity	fMRI	Motor FND including FS with mixed psychiatric conditions and concomitant medication use	• Diez et al. (2019) • Diez et al. (2021)
Temporoparietal junction	↑ Connectivity to the insula with symptom severity ↓ Connectivity between the right TPJ and the sensorimotor cortex, cerebellar vermis, bilateral SMA, and right insula	fMRI	Motor FND including FS with mixed psychiatric conditions and concomitant medication use	• Diez et al. (2019) • Maurer et al. (2016)

ACC: anterior cingulate cortex; dlPFC: dorsolateral prefrontal cortex; DMN: default mode network; dmPFC: dorsomedial prefrontal cortex; DTI: diffusion tensor imaging; fMRI: functional magnetic resonance imaging; FND: functional neurological disorders; FS: functional seizure; MCC: middle cingulate cortex; MRI: magnetic resonance imaging; PET: positron emission tomography; PFC: prefrontal cortex; SMA: supplementary motor area; SPECT: single photon emission computed tomography; TBI: traumatic brain injury; TPJ: temporoparietal junction.

### A complexity science perspective on functional seizure mechanisms

This heterogeneity may be reconciled when viewed through the lens of complexity science, an interdisciplinary field that seeks to characterize the common properties and behavior of complex systems of dynamically interacting parts, from brains to ecosystems, to stock markets (Olthof et al., 2023; Turkheimer et al., 2021). Complexity science advances a distributed, interactional, and dynamic account of brain function, wherein behavior and cognition are the result of dynamic interactions spanning the brain as a whole.

From this perspective, the neural pathophysiology associated with FS is conceptualized as a dynamic, whole-brain phenomenon. Accordingly, the manifestation of FS for a given patient at a given time—contingent on the individual’s predisposing, precipitating, and perpetuating factors—will arise as a relatively idiosyncratic set of dynamic brain network interactions. By applying complex systems statistical approaches, assessments can abstract from a search for a representative pattern of connectivity changes to a search for commonalities in the dynamic traversal of whole-brain patterns over time. The particular large-scale network patterns, the dynamics of the switching between them, and their relative frequency of occurrence would be assumed to vary across time for a given individual (e.g., based on disease course) and across individuals—naturally leading to heterogeneity when comparing cross-sectional group-averaged findings. In this view, for example, predisposition to FS in some patients may be associated with a mode of brain function that is strongly driven by ascending autonomic inputs and endocrine responses related to hyperarousal/stress. Earlier stages might feature a preponderance of patterns of heightened connectivity between limbic and insular regions and other cortical regions in the broader context of a dynamic interplay between ascending emotional-viscerosomatic inputs and descending top-down inhibition via the prefrontal cortex. Subsequently, over time, the likelihood of FS onset may progressively increase as the nervous system is overwhelmed, resulting in excessive top-down inhibition, which, in turn, precipitates dysregulation in other cortical regions such as somatomotor and posterior parietal (e.g., right temporoparietal junction) cortex ultimately triggering a (learned) seizure motor schema. This dynamic systems-level account is broadly consistent with past findings of top-down alterations in FND (Hallett et al., 2022) and the role of predictive processing (Drane et al., 2020; Edwards et al., 2012). It is also supported by research indicating that patients with FND typically have a history of early adverse childhood experiences and consequent chronic hyperarousal/hypervigilance, along with increased coupling of amygdalar-motor and insular-motor cortices compared to controls, which itself suggests the development of maladaptive connections over time (Diez et al., 2021).

Overall, adopting a complexity science perspective allows heterogeneity in cross-sectional group-level findings to be seen as a potential feature—and not a bug—of the neurobiology of FS. It also provides a scheme to understand the inherently multifocal and distributed nature of the underlying pathophysiology. This approach has been proposed to explain similar clinical and functional neuroimaging discrepancies arising out of the mechanistic psychedelic literature (Girn et al., 2023; Hipólito et al., 2023)—discussed more below.

In summary, FS involve complex interactions across biological, psychological, and social domains. The ICM is the leading model for understanding FS, implicating pathologic neuropsychological constructs spanning multiple large-scale brain networks. Studies have identified particular dysfunctional networks, but inconsistent findings across investigations have precluded the development of unified mechanistic models; hypo- or hyper-activation of the rTPJ, increases and decreases networks related to emotion and motor networks, attentional systems and motor planning systems, and the default mode network (Table 1). The complex systems account described above may help facilitate such mechanistic models, wherein predisposing, precipitating, and perpetuating factors for FS can be understood as a dynamic whole-brain process aimed at coping with chronic sympathetic nervous system/stress hyperactivity. This idea aligns with the mechanistic direction proposed by Girn et al. (2023), regarding the action of psychedelics on large-scale brain network dynamics as described in the next section.

## Psychedelic mechanism of action: From molecule to behavior

The mechanism of action of psychedelics is an active area of research. As the research unfolds, the general neurological framework has begun to develop wherein psychedelics act upon different molecular targets to promote structural and functional plasticity across spatial scales. At the micro/mesoscale, this corresponds to a disruption in the coordinated oscillatory firing of neuronal populations—particularly in the prefrontal cortex for serotonergic psychedelics and ketamine (McMillan and Muthukumaraswamy, 2020; Muthukumaraswamy et al., 2013, 2015; Schartner et al., 2017). At the macroscale, these disruptions result in altered patterns of FC within and between multiple large-scale networks and alterations in the dynamics of this connectivity over time (Girn et al., 2023). These functional changes are underpinned and facilitated by structural neuroplastic (Calder and Hasler, 2023)— and “metaplastic” effects (Nardou et al., 2019, 2023)—thought to be central to the putative ability for this class of drugs to induce lasting symptom reductions across several mental health conditions, including depression, end-of-life distress, anxiety disorders, PTSD, and substance use disorders (Table 1).

### Molecular mechanisms of psychedelics

Multiple molecular targets and downstream signaling cascades have been identified, with some overlap across drugs (Inserra et al., 2021). Classic psychedelics are known to elicit their primary neural and subjective effects via partial agonism of the 5-HT2A receptor—an excitatory G-protein coupled receptor that has the net effect of increasing neuronal excitability via increased glutamatergic neurotransmission. By contrast, ketamine’s antidepressant effects have been primarily linked to NMDA receptor antagonism and, in particular, glutamatergic disinhibition in the prefrontal cortex. Despite these differences, both classic psychedelics and ketamine converge on the brain-derived neurotrophic factor-TrkB intracellular signaling pathway. This pathway serves to activate mTOR and induces neuroplastic effects via changes in gene expression that support increased dendritogenesis and synaptogenesis (de Vos et al., 2021). MDMA exhibits a relatively distinct neuropharmacological profile but shares with serotonergic psychedelics the capacity to activate the 5-HT2A receptor (upstream of increased glutamate release)—in this case, primarily via the upregulation of endogenous 5-HT, but also to a lesser degree via direct affinity for this receptor (de la Torre et al., 2004). Theoretical models have highlighted the pro-plasticity effects of these compounds as central to their therapeutic efficacy (Brouwer and Carhart-Harris, 2021; Nardou et al., 2023). Recent evidence suggests that each of these drugs may produce lasting increases in both “metaplasticity” and “hyperplasticity,” potentially at distinct time scales. Metaplasticity corresponds to an increase in the ease with which neuroplasticity (e.g., long-term potentiation) can be induced via non-drug inputs (Dankovich and Rizzoli, 2022; Dityatev and Schachner, 2003; Dityatev et al., 2010; Nardou et al., 2023), whereas “hyperplasticity” corresponds to the ability to directly induce patterns of neuroplastic change.

### Neuroimaging and complexity science mechanisms of psychedelics

Human neuroimaging studies of the acute psychedelic experience afford a translational assessment of the macroscale neural consequences of the effects observed at the micro/mesoscale. We focus here on results pertaining to functional magnetic resonance imaging (fMRI) given that this modality comprises the majority of psychedelic neuroimaging research, and because this allows comparisons with fMRI research on FS. fMRI investigations of the acute psychedelic experience have demonstrated a complex set of FC alterations spanning nearly all large-scale brain networks (McCulloch et al., 2022). Broadly, findings suggest that psychedelics can reduce the functional segregation of large-scale brain networks, leading to more interconnected and flexible or entropic brain connectivity and dynamics (Table 2). Beyond these general effects, however, the psychedelic functional neuroimaging literature—similar to what has been observed for FS—contains heterogeneous findings and inconsistencies (Girn et al., 2023). In particular, the specific pattern of changes between large-scale networks has exhibited poor overlap across studies, and changes within large-scale networks (e.g., default mode network disintegration) have been found for a variety of non-psychedelic drugs and have not been reliably mapped to subjective effects (Girn et al., 2023; Müller et al., 2018, 2021).

**Table 2.** Psychedelic-induced brain region and network alterations shared with FS.

Brain region	Key findings	Methods	Cohort	Studies
DMN	↑Functional connectivity to various areas including emotional, attentional, and sensorimotor brain areas ↑ Functional connectivity between insula and TPJ ↓ Functional connectivity within the DMN	ASL, BOLD, fMRI, MEG, EEG-fMRI	Healthy volunteers	• Carhart-Harris et al. (2016) • Preller et al. (2019) • Preller et al. (2020) • Carhart-Harris et al. (2012) • Tagliazucchi et al. (2016) • Girn et al. (2022) • Timmermann et al. (2023) • Roseman et al. (2014)
TPJ	↑ Bilateral functional connectivity ↑ Functional connectivity between TPJ and sensorimotor cortex	fMRI	Healthy volunteers	• Tagliazucchi et al. (2016)
Medial temporal	↑ Functional connectivity in the occipital cortex, superior temporal gyrus, and postcentral gyrus, and precuneus. ↓ Functional connectivity with medial PFC and lateral PFC, the cingulum, the insula, and the temporoparietal junction ↓ Right amygdala activation to negative and neutral pictures ↓ Functional connectivity with posterior cingulate/retrosplenial cortex	ASL, BOLD, fMRI, MEG,	Healthy volunteers	• Preller et al. (2018) • Kraehenmann et al. (2015) • Carhart-Harris et al. (2016)
Motor cortex	↓ Functional connectivity within the somatomotor network ↑ Functional connectivity with the DMN and frontoparietal network	fMRI EEG-fMRI	Healthy volunteers	• Tagliazucchi et al. (2016) • Girn et al. (2022) • Timmermann et al. (2023) • Roseman et al. (2014)

ASL: arterial spin labeling; BOLD: blood oxygen level dependent; DMN: default mode network; EEG: electroencephalogram; fMRI: functional magnetic resonance imaging; MEG: magnetoencephalography; PFC: prefrontal cortex; TPJ: temporoparietal junction.

Of note, Girn et al. (2023) proposed a framework that describes psychedelics as best seen as inducing a distinct *mode* of brain function that exhibits a particular set of dynamical properties in terms of its spontaneous and stimulus-evoked activity. This framework proposes that the brain, under psychedelics, becomes less constrained and more easily able to explore a greater dynamic range of states, and exhibits an increased sensitivity to environmental stimuli and enhanced ability to respond to inputs adaptively and flexibly. These properties manifest in a dosing-session-specific way depending on internal (i.e., personality, beliefs, expectations, ongoing cognitive-emotional state) and external (physical and social environment) factors, thereby resulting in significant inter-individual and inter-study variability in subjective effects. Given close mappings between subjective experience and brain function, this would result in relatively idiosyncratic and time-varying neural connectivity when measured by functional neuroimaging modalities such as fMRI. Accordingly, the framework posits that complex system analyses aimed at assessing the dynamic traversal of distinct brain states and quantitative properties thereof, rather than searching for a canonical connectivity pattern, may help reconcile and unify past findings (Girn et al., 2023). A variety of analytical approaches to characterizing brain state dynamics have been proposed in the neuroimaging literature (Lurie et al., 2020), and several have been applied to acute psychedelic data (Atasoy et al., 2018; Lord et al., 2019; Olsen et al., 2022; Singleton et al., 2022). Broadly, the central idea is to identify distinct patterns of interregional or internetwork FC that recur (discretely or in a graded fashion) over the course of a given fMRI or EEG session (usually ~6–10 min). This approach is in contrast to averaging across a given session to find the “static” central tendency. Relevant dynamic metrics can be assessed cross-sectionally and/or longitudinally and could include, for example, the number and precise topography of identified states, their switching frequency and inter-state transition probabilities, and the predictability or orderliness of their dynamics over time (or, conversely, their entropy). The revealed states and their metrics can be ascertained at varying spatial and temporal scales, depending on the hypotheses in question.

The current body of literature on the mechanisms of psychedelics suggests intricate and extensive interactions within the brain across multiple spatial scales, spanning a variety of regions and large-scale networks (Table 2). Complex system analysis may help identify changes to whole-brain functional dynamics that can unify seemingly inconsistent region or network-specific psychedelic neuroimaging findings, similar to the methodology that could be applied to reconcile the discrepancies in the FS literature. This novel mechanistic lens could provide a basis for exploring the potential overlap between the underlying mechanisms of psychedelics and FS. In particular, it raises the question of whether the ability of psychedelics to transiently induce a dynamic and flexible mode of brain function may be apt to disrupt the progressive network dysregulation that characterizes FS. In addition, complex system analyses may assist in the development of treatment strategies and evaluating the outcomes of psychedelic therapy interventions in the context of FS.

## PAT as a treatment for FS

### Overview of PAT

PAT is a therapeutic intervention that consists of administering a psychedelic substance in a carefully controlled environment with preparatory and follow-up psychological support centered around the acute experience (Schenberg, 2018). In this way, PAT represents a combined pharmaco- and psychotherapy approach that leverages both the acute psycho-emotional experience elicited by the drug, as well as its potentially experience-agnostic neurobiological effects, toward lasting positive change in behavior and well-being (Greenway et al., 2020). Evidence suggests that PAT can generate profound psychosocial changes, leading to marked symptom reductions across several mental disorders (Andersen et al., 2021) (Table 1). The fact that PAT acts upon biological, psychosocial, and environmental factors suggests its potential to serve as an innovative adjunct treatment for FS. Indeed, the biopsychosocial model—an expansion of the long-hegemonic biomedical model—has facilitated our understanding of how psychedelics work and has also been applied to FS, serving as a basis for the clinical model of the ICM in practice. PAT may help alleviate FS symptoms either as a result of addressing comorbid mental health conditions and/or by influencing associated brain networks and network dynamics which are causative of the condition—in a manner not dissimilar to what has been proposed for chronic pain (Castellanos et al., 2020).

Supporting the potential efficacy of PAT for FS, a recent case report by our group described significant improvements in a patient’s FS, as well as comorbid depression and PTSD. Specifically, after unsuccessful treatment attempts, the patient received 3 weeks of standardized KAT (three preparatory sessions, three ketamine sessions with sublingual and intranasal administration, and three integration sessions the day after dosing), followed by 20 weeks of ketamine maintenance with integrative psychotherapy including CBT, psychodynamic therapy, and mindfulness approaches. After KAT, the patient experienced substantial improvements in functional seizure frequency and severity, as well as Quick Inventory of Depressive Symptomatology (QIDS) and Work and Social Adjustment Scale (WSAS) scores representing improvements in depression, day-to-day quality of life, and function. Notably, the patient was able to taper off of benzodiazepines and described that the KAT sessions allowed her to process and access trauma without triggering a seizure (Argento et al., 2023).

We propose that FS offer a well-defined cohort for investigating the underlying mechanisms of psychedelics and their potential as a novel treatment for FND. Considering this proposition for a future clinical trial, we outline an adaptable framework below. This framework involves examining the therapeutic effects of psychedelics in FS patients, leveraging complex systems analysis, and utilizing pre- and post-treatment assessments to elucidate the mechanisms involved. By employing this framework, we can advance our understanding of the mechanisms of psychedelic action via analysis of their effect in FND, thereby paving the way for innovative treatments in this challenging clinical domain.

### FS as a defined cohort

FS patients form a relatively homogenous clinical group, as they can be easily diagnosed and distinguished from epileptic seizures under appropriate conditions. Trained neurologists and epileptologists possess the expertise to differentiate between functional and epileptic seizures based on semiology, with distinct features outlined in the literature (LaFrance et al., 2013a). Furthermore, the use of video EEG serves as an objective marker, allowing for a high level of certainty in distinguishing FS from epileptic seizures (LaFrance et al., 2013b).

The ability to objectively define FS patients renders this population a clinically well-defined cohort to assess outcomes for future clinical psychedelic trials. The increased precision in patient selection would enhance the internal validity of the study, ensuring that the observed effects of psychedelic therapy can be more confidently attributed to the treatment itself rather than confounding factors related to patient heterogeneity.

### Measuring functional seizure clinical outcomes

Reduction in seizure frequency is a typical clinical measure of therapeutic success in FS trials. Decreased seizure frequency correlates with improvements in functional capacity and comorbid mental health conditions serving as secondary outcome measures of the effectiveness of the intervention.

Seizure frequency provides an easily measurable, direct quantitative variable that can be assessed both objectively by the individual experiencing the seizures and by others involved in their care. This objective measure complements the subjective experiences reported by the individuals themselves regarding their seizures. In comparison to other psychedelic therapy trials that primarily evaluate changes in mental health conditions, assessing seizure frequency as a primary outcome measure offers a more reliable and quantifiable indicator of clinical success.

Furthermore, the use of seizure frequency as a primary outcome measure allows for a more reliable separation of responders to the psychedelic therapy intervention for subsequent analysis. This would enable researchers to identify and analyze the specific subgroup of patients who demonstrate a favorable response.

### Applying complex systems perspectives

The models proposed to explain the mechanisms of action of psychedelics and the development of FS suggest that they both involve distributed and multifocal neural alterations within and across large-scale brain networks, implying complex and dynamic interactions. Functional neuroimaging studies have thus far identified aberrant network connectivity supporting these models. However, findings across different studies have yielded heterogeneous and seemingly inconsistent results.

We posit that a complex system perspective holds the potential to reconcile such apparent discrepancies. This perspective seeks to identify dynamic whole-brain changes and properties thereof, explicitly highlighting temporal (i.e., across the disease course) and inter-individual heterogeneity as a core feature of the underlying neurobiology. For example, in the case of FS, the predisposing factors of chronic stress and hyperarousal may lead to a whole-brain dynamical regime that is strongly influenced by a need to monitor, attenuate, and respond to strong ascending autonomic inputs. This may manifest in relatively person-specific dynamic patterns of dysfunction between emotional, cognitive, and motor networks. However, the commonality in the face of this heterogeneity is that global brain dynamics are functioning in a coordinated manner toward a particular goal, with shared points of vulnerability. Complex system analyses may therefore facilitate novel insights into the overall gestalt of neural alterations underpinning FS. Moreover, as mentioned, this suggests relevant mechanistic overlap with psychedelics—which have been construed as altering fundamental properties of global brain dynamics that may help “reset” or provide a therapeutically relevant deviation from dysfunctional patterns of functioning. As such, by applying a complex system approach, a deeper understanding of the therapeutic mechanisms can be achieved. For example, neuroimaging investigations of FS could apply analyses derived from complexity science which assess system-level properties such as modularity, entropy, metastability, and criticality (Hancock et al., 2022a, 2022b; Wilting and Priesemann, 2019)—as have been fruitfully applied to characterize psychedelic effects (Carhart-Harris et al., 2014, 2018; Daws et al., 2022; Girn et al., 2023; Toker et al., 2022), as well as other mental states and conditions (Hancock et al., 2023; Turkheimer et al., 2021).

FS offer a unique opportunity to apply complex systems analysis in unraveling the therapeutic mechanisms of psychedelics. The ability to clearly define FS patients and separate treatment responsiveness generates a well-defined cohort to examine whether complex systems analysis can elucidate the mechanistic therapeutic effects of psychedelics. Moreover, the findings that arise by employing complex systems analyses of functional imaging from a population of FS patients before and after PAT could reliably inform on (i) the pre-treatment imaging characteristics that respond to PAT intervention, (ii) the post-treatment imaging features serving as potential markers of treatment success, and (iii) the changes in dynamic brain interactions from pre to post-treatment, shedding light on the mechanism by which psychedelics work to treat FS and, potentially, FND as a whole.

## Conclusion

In conclusion, we propose FS as a well-defined cohort to study the potential for complex system analysis to expand our mechanistic understanding of psychedelics’ neural and psychosocial effects. The appropriateness of utilizing FS for this pursuit stems from the precisely defined diagnostic attributes of this unique population, along with the ability to discern responses based on a dichotomous, quantifiable outcome linked to seizure reduction. Furthermore, this choice is underscored by the potential alignment between PAT’s therapeutic mechanisms and the intricate neuropsychological dysfunction that underlies FS.

Moreover, the complex system approach highlighted in this paper could facilitate the development of a compelling mechanistic model and predictive biomarkers for FS pathophysiology and its resolution. This potential lies in shifting the focus from targeting specific brain networks to restoring neural flexibility and diversity through increased global integration relaxing compromised top-down cortical inhibitory processes that facilitate an unlearning of the seizure scaffold. Consequently, this approach reduces the need to target inter-individual differences, allowing for more generalized, accessible, and, perhaps, more effective treatment across FS phenotypes.

A future trial exploring complex system analyses in FS patients treated with PAT could uncover crucial correlations, deepening our understanding of psychedelic mechanisms and opening the door for potential treatment options in the typically highly treatment-resistant conditions of FS and FND. Importantly, this endeavor serves as a blueprint that, applied to other neurologic conditions, bridges clinical neurology with psychedelic medicine paving the way for the emergence of the field of psychedelic neurology.

## Supplemental Material

sj-docx-1-jop-10.1177_02698811241248395 – Supplemental material for Exploring psychedelic-assisted therapy in the treatment of functional seizures: A review of underlying mechanisms and associated brain networks Supplemental material, sj-docx-1-jop-10.1177_02698811241248395 for Exploring psychedelic-assisted therapy in the treatment of functional seizures: A review of underlying mechanisms and associated brain networks by Evan Cole Lewis, Alexandria Jaeger, Manesh Girn, Egiroh Omene, Madeline Brendle and Elena Argento in Journal of Psychopharmacology
