# Supplementary material for: Exploring psychedelic-assisted therapy in the treatment of functional seizures: A review of underlying mechanisms and associated brain networks
Source: J Psychopharmacol. 2024 Apr 23;38(5):407–16. doi: 10.1177/02698811241248395 (PMC11102649; doi:10.1177/02698811241248395)
Supplement: sj-docx-1-jop-10.1177_02698811241248395 – Supplemental material for Exploring psychedelic-assisted therapy in the treatment of functional seizures: A review of underlying mechanisms and associated brain networks [file sj-docx-1-jop-10.1177_02698811241248395.docx]

**Supplemental Material**

| Table 1 Selected psychedelic-assisted therapy trials for psychiatric disorders | | | | | | |
| --- | --- | --- | --- | --- | --- | --- |
| Studies | **Substance** | **Design** | **Dose** | **Indication** | **Phase** | **Key findings** |
| (Mitchell et al., 2021) | MDMA | RCT, double-blind, multi-site, 3 PAT sessions, n=94 | 80 – 120mg | PTSD | 3 | 86.5% of people had a clinically significant decrease in PTSD as assessed by **CAPS-5** and **SDS**, 71.2% of participants no longer met criteria for PTSD by end of study (as compared to 47.6% who received placebo). |
| (Wolfson et al., 2020) | MDMA | RCT, double-blind, crossover, 2 PAT sessions, n=18 | 125-187.5 mg | End of life distress | 2 | The MDMA group (Δ-23.5) compared to placebo (Δ-8.8) trended with a reduced anxiety with **STAI-Trait** score at one-month post treatment but was not statistically significant. The MDMA group (Δ12.9; Δ0.4) was significantly increased in growth and mindfulness compared to placebo group (Δ-2.6; Δ0.2) with the **PTGI** and **FFMQ** respectfully at one month post treatment. The crossover results showed reduced anxiety and depression through the 12-month follow up via **STAI** **BDI-II**, and **MADRS** scores. |
| (Danforth et al., 2018) | MDMA | RCT, double blind, 2 PAT sessions n=12 | 75 – 125mg | Social anxiety in ASD | 3 | A statistically significant reduction in anxiety that persisted to the 6-month follow up was observed by **LSAS** compared to control. |
| (Goodwin et al., 2022) | Psilocybin | RCT, double blind, 1 PAT session n=233 | 10mg or 25mg | MDD | 2 | A 21.4% remission rate of depression for the 25mg assessed by the **MADRS**, a significant change from both the 10mg group and 1 mg placebo group. |
| (Carhart-Harris et al., 2021) | Psilocybin | RCT, double-blind, n=59, 2 PAT sessions, compared to escitalopram | 25mg | MDD | 2 | No significant difference in change in **QIDS-SR16** scores when comparing treatment arms, 70/57% response/remission rates in psilocybin arm vs. 57/28% in escitalopram arm at 6-week follow-up, most secondary analyses favored psilocybin arm |
| (Griffiths et al., 2016) | Psilocybin | RCT, double-blind, crossover, active placebo (low-dose psilocybin 1-3mg/70kg), 1 PAT session, n=51 | 22 – 30mg /70kg | End of life distress | 2 | **GRID-HAMD-17** depression scores: 92% response and 60% remission at 5 weeks in treatment group vs. 32% and 16% for active-placebo, 79% persistent at 6 months. **HAM-A** anxiety scores were 76% response and 52% remission at 5 weeks in treatment group vs. 24% and 12% for active-placebo, 83% persistent at 6 months, outcomes correlated with subjective experience ratings |
| (Moreno et al., 2006) | Psilocybin | Modified double-blind, 4 PAT starting with low dose then randomized 3 testing dose, Open Label, n=9 | (control) 25mg/kg and (testing) 100mg/kg and 200 mg/kg and 300mg/kg | OCD | 1 | Significant decrease in OCD symptoms and **YBOCS** score that lasted past 24-hours for all testing doses, but no significant effect of dose on **YBOCS** scores. |
| (Gasser et al., 2015) | LSD | RCT, double-blind, crossover, 2 PAT session, n=12 | 200µg | End of life distress | 2 | A significant reduction in anxiety by **STAI** scores was observed with LSD (Δ-44.1) compared to placebo (Δ-19.3) that persisted at the 12 month follow up. |
| (Phillips et al., 2020) | Ketamine | Secondary analysis, RCT, double-blind crossover, n=37 | 0.5 mg/kg | Treatment resistant depression |  | Suicidality decreased in participants by **MADRS-SI** score of 1.7 points at seven days post infusion, 2.3-point decrease from after six ketamine infusions, with no additional significant reduction or relapse at 10 ketamine infusions |
| (Shiroma et al., 2020) | Ketamine | RCT, double-blind, n=54 , adolescents age 13-17, 6 infusions vs 1 infusion | 0.5 mg/kg | MDD | 1 | Significant reduction in MDD with both single and six ketamine infusions. No significant difference in MDD reduction or depression relapse by **MADRS** score between single (mean change = 21) vs six (mean change = 17.2) ketamine treatments. |
| (Dwyer et al., 2021) | Ketamine | RCT, double-blind crossover, n= 17, single infusion | 0.5 mg/kg | MDD | 1 | Single ketamine infusion significantly reduced the after 24hrs and 14 days, 77% of participants responded to ketamine as compared to 35% of participants responded to control midazolam, as measured by **MADRS** score |

**Table. S1.** Abbreviations: Randomized Clinical Trial (RCT); post-traumatic stress disorder (PTSD); Psychedelic-assisted Therapy (PAT); autism spectrum disorder (ASD); major depressive disorder (MDD); Obsessive Compulsive Disorder (OCD); Montgomery–Åsberg Depression Rating Scale score (MADRS); MADRS suicide item (MADRS-SI); State-Trait Anxiety Inventory (STAI); Post Traumatic Growth Inventory (PTGI); Five Factor Mindfulness Questionnaire (FFMQ); Beck Depression Inventory-II (BDI-II); The Yale-Brown Obsessive Compulsive Scale (YBOCS); Leibowitz Social Anxiety Scale (LSAS)

**References**

Carhart-Harris RL, Giribaldi B, Watts R, et al. (2021) Trial of Psilocybin versus Escitalopram for Depression. *N Engl J Med* 384(15): 1402-1411.

Danforth AL, Grob CS, Struble C, et al. (2018) Reduction in social anxiety after MDMA-assisted psychotherapy with autistic adults: a randomized, double-blind, placebo-controlled pilot study. *Psychopharmacology* 235(11): 3137-3148.

Dwyer JB, Landeros-Weisenberger A, Johnson JA, et al. (2021) Efficacy of Intravenous Ketamine in Adolescent Treatment-Resistant Depression: A Randomized Midazolam-Controlled Trial. *American Journal of Psychiatry* 178(4): 352-362.

Gasser P, Kirchner K and Passie T (2015) LSD-assisted psychotherapy for anxiety associated with a life-threatening disease: a qualitative study of acute and sustained subjective effects. *J Psychopharmacol* 29(1): 57-68.

Goodwin GM, Aaronson ST, Alvarez O, et al. (2022) Single-Dose Psilocybin for a Treatment-Resistant Episode of Major Depression. *N Engl J Med* 387(18): 1637-1648.

Griffiths RR, Johnson MW, Carducci MA, et al. (2016) Psilocybin produces substantial and sustained decreases in depression and anxiety in patients with life-threatening cancer: A randomized double-blind trial. *J Psychopharmacol* 30(12): 1181-1197.

Mitchell JM, Bogenschutz M, Lilienstein A, et al. (2021) MDMA-assisted therapy for severe PTSD: a randomized, double-blind, placebo-controlled phase 3 study. *Nature Medicine* 27(6): 1025-1033.

Moreno FA, Wiegand CB, Taitano EK, et al. (2006) Safety, tolerability, and efficacy of psilocybin in 9 patients with obsessive-compulsive disorder. *Journal of Clinical Psychiatry* 67(11): 1735-1740.

Phillips JL, Norris S, Talbot J, et al. (2020) Single and repeated ketamine infusions for reduction of suicidal ideation in treatment-resistant depression. *Neuropsychopharmacology* 45(4): 606-612.

Shiroma PR, Thuras P, Wels J, et al. (2020) A randomized, double-blind, active placebo-controlled study of efficacy, safety, and durability of repeated vs single subanesthetic ketamine for treatment-resistant depression. *Translational Psychiatry* 10(1): 206.

Wolfson PE, Andries J, Feduccia AA, et al. (2020) MDMA-assisted psychotherapy for treatment of anxiety and other psychological distress related to life-threatening illnesses: a randomized pilot study. *Sci Rep* 10(1): 20442.
